# Atomically sculptured heart in oxide film using convergent electron beam

**DOI:** 10.1186/s42649-020-00050-7

**Published:** 2021-01-11

**Authors:** Gwangyeob Lee, Seung-Hyub Baek, Hye Jung Chang

**Affiliations:** 1 Advanced Analysis Center, Korea Institute of Science and Technology, Seoul, 02792 Republic of Korea; 2 Electronic Materials Research Center, Korea Institute of Science and Technology, Seoul, 02792 Republic of Korea; 3 Division of Nano and Information Technology, KIST School, University of Science and Technology, Seoul, 02792 Republic of Korea

**Keywords:** Epitaxial crystallization, Electron beam irradiation, Spherical aberration corrected scanning transmission electron microscope, Nanoarchitectonics

## Abstract

We demonstrate a fabrication of an atomically controlled single-crystal heart-shaped nanostructure using a convergent electron beam in a scanning transmission electron microscope. The delicately controlled e-beam enable epitaxial crystallization of perovskite oxide LaAlO_3_ grown out of the relative conductive interface (i.e. 2 dimensional electron gas) between amorphous LaAlO_3_/crystalline SrTiO_3_.

## Description

Lithography techniques utilizing various sources including light, X-rays, electron beams (e-beams), and ion beams have been investigated to obtain better performance (Levenson et al. 1982; Ehrfeld and Lehr 1995; Watt et al. 2005). Among these techniques, e-beam lithography is one of the most promising methods of fabricating nanostructures because of its excellent spatial resolution (Tseng et al. 2003; Altissimo 2010). Atomically controlled nanostructure sculpting can be conducted using recent advances in aberration-corrected scanning transmission electron microscopy (STEM) (Song et al. 2011; Jesse et al. 2015). In this study, we investigated the e-beam controlled epitaxial crystallization of an amorphous LaAlO_3_ (a-LAO) thin film that had a conductive interface with a SrTiO_3_ (STO) substrate (Moon et al. 2016).

The a-LAO thin film was grown on TiO_2_-terminated STO substrates at room temperature by pulsed laser deposition in an oxygen atmosphere. Cross-sectional TEM specimens were prepared by standard mechanical polishing (Struers; Labopol-5) and subsequent argon-ion milling (PIPS 691; Gatan). Observation of the crystallization of the a-LAO under e-beam irradiation was performed using aberration-corrected STEM (Titan S80–300; FEI), and the convergent e-beam was controlled by STEM software. The acceleration voltage and dose rate of the incident e-beam were 300 keV and 0.169 × 10^9^ e^−^ A^− 2^ s^− 1^, respectively.

**Fig. 1:**
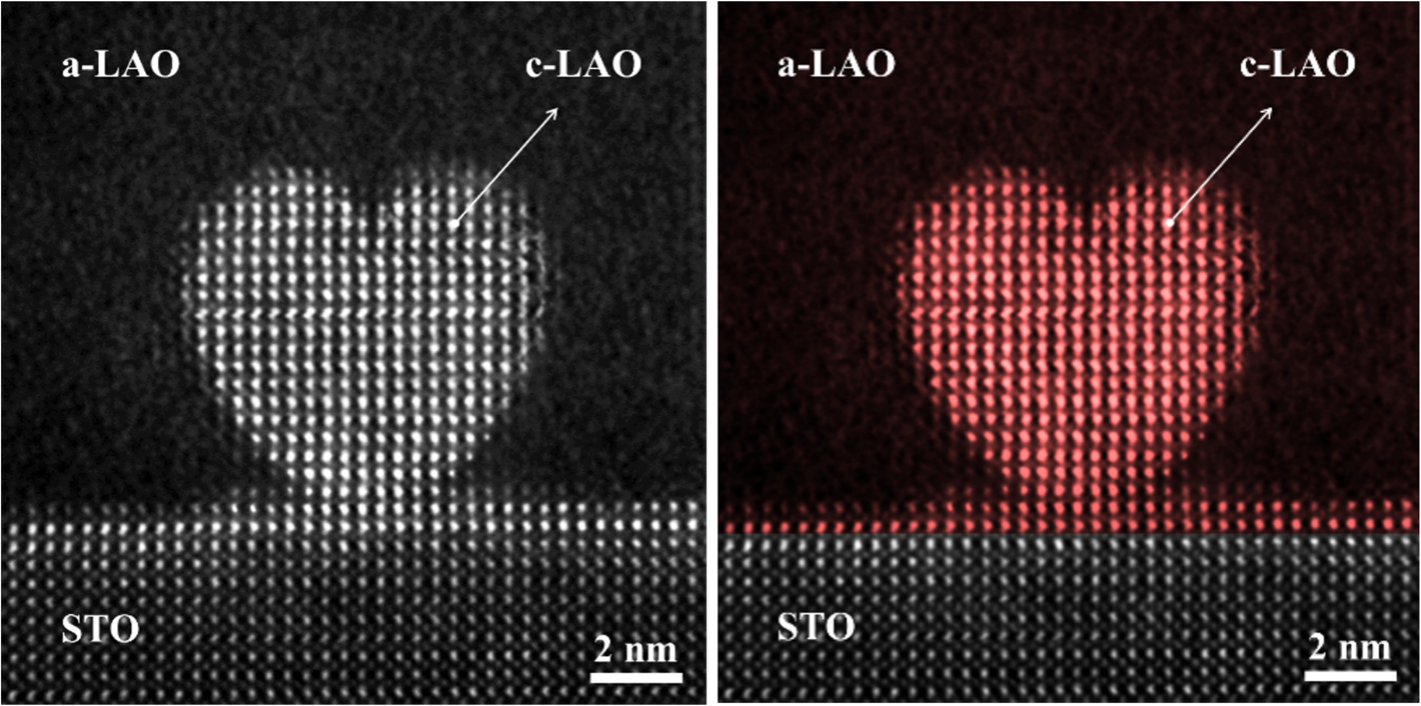
A high-angle annular dark-field scanning transmission electron microscopy (HAADF STEM) (left) and colored image (right) of the sculptured heart-shaped crystallized LAO in the a-LAO region through e-beam irradiation in STEM

Under delicately controlled e-beam irradiation, the amorphous structure changed into a crystalline structure with epitaxy with the STO substrate. Using this technique, we sculptured heart-shaped crystallized LAO in a-LAO layer (Fig. 1). The atomically controlled nanostructure sculpting was conducted using several control parameters such as the interfacial conductivity, dose rate, and e-beam’s distance from the heterointerface (Lee et al. 2017). The heart-shaped crystallized region with a brighter contrast in the high-angle annular dark-field (HAADF) STEM image was perovskite-type pseudocubic LAO, which was confirmed by the chemical composition and diffraction pattern analyses (Lee et al. 2017). We hope that e-beam lithography using sub-nano scale e-beams in STEM can be applied to manipulate the structures and properties of materials and devices.

## Data Availability

Not applicable.
